# Intestinal gene expression in heat-stressed broilers selected for high water efficiency

**DOI:** 10.3389/fphys.2025.1609065

**Published:** 2026-05-13

**Authors:** Kentu Lassiter, Loujain Aloui, Elizabeth S. Greene, Marciela Maqaeda, Kirsten Schaeffer, Brooklee Roach, Travis Tabler, Robert F. Wideman, Sara Orlowski, Sami Dridi, Walter G. Bottje

**Affiliations:** 1 Center of Excellence for Poultry Science, Division of Agriculture, University of Arkansas, Fayetteville, AR, United States; 2 Higher School of Agriculture of Mograne, University of Carthage, Zaghouan, Tunisia

**Keywords:** broilers, gene expression, heat stress, intestines, water use efficiency

## Abstract

**Background:**

The major goal of this study was to compare mRNA expression associated with water homeostasis in intestines of high water efficient (HWE) broilers to expression in unselected Modern Random Bred (MRB) broilers under thermoneutral (TN) and heat stress (HS) environments.

**Methods:**

A 2 × 2 factorial study was conducted with two lines (MRB and HWE) and environments (TN and HS). Males were raised in environmental chambers and maintained in TN conditions from 0 to 4 weeks. From 4 to 7 weeks, broilers were maintained under TN conditions (25C) or exposed to cyclic HS conditions (35C, 8 h/d). At 7 weeks, upper and lower duodenum, and cecum samples were obtained. mRNA was determined by RT-PCR for; 1) heat shock proteins (HSP70, HSP90), 2) the arginine vasotocin (AVT), mesotocin (MT) and receptors (VR2, VR1a, VR1b, MTR), 3) the renin angiotensin system (RAS): angiotensinogen (AGT), angiotensin receptors, (AT1, AT2), renin (REN), AGT converting enzyme (ACE), and Na^+^/^+^ATPase (ATP1B1), 4) aquaporins (AQP1-5 and 11), and 5) tight junction proteins occludin (OCLN), and claudins (CLDN2, CLDN15).

**Results:**

A main effect of HS was observed for HSP70 and HSP90 expression in all three intestinal-segments. In lower duodenum, HS-induced upregulation of AVT was accompanied by elevations in VR2, VR1a, VRb, AT1, AQPs (1, 3, 11) OCLN, and CLND2, CLDN15. Main effects of broiler line were observed with HWE exhibiting increased mRNA expression of VR2, VR1a, VR1b, AQP1, AQP3 and AQP5 and CLDN2 in the upper duodenum. There was also higher VR2, MT and AQP5 and lower expression of CLDN15 in the lower duodenum but there were no differences in gene expression between MRB and HWE lines in the cecum. Heat stress upregulated AVT, VR1a, MTR, AVT/MT ratio, AQP1, and AQP3 but downregulated ACE in the upper duodenum. Heat stress also upregulated AVT, VR2, VR1a, VR1b, MTR, AT1, AT2 AQP3, ACP5, OCLN, CLDN2, CLDN15 in the lower duodenum and cecum.

**Conclusion:**

The results provide insight into mechanisms responsible for water efficiency in HWE broilers and provide new information in gene expression in the gastrointestinal tract of broilers exposed to chronic cyclic heat stress conditions.

## 1 Introduction

The last decade has been the hottest on record since global temperatures have been recorded (NASA, 2025). Currently, record high temperatures are being set once again in the United States (US cities face record high temperatures on worst day of heat wave). Droughts are increasing incidents of water scarcity and placing water availability in jeopardy for billions of people worldwide. It is expected that extreme temperatures will be higher and last for longer time periods (Xu et al., 2018; Tollefson, 2022) with devasting effects on global crop production (Liu et al., 2022). Water scarcity is a major concern for intensive poultry production (El Sabry et al., 2023). Thus, water footprint will become increasingly important. Although there has been a great deal of effort made in commercial broiler genetics to improve traits such as liveability and feed efficiency (feed conversion ratio, FCR), no sustained effort in selection of broilers for water efficiency has occurred. For this reason, divergent selection of broilers for high and low water conversion ratio (WCR, calculated as weight intake per body weight gain) was initiated (Hilts, 2021; Hiltz et al., 2021). We have determined that selecting for WCR has not negatively impacted growth performance when compared to the original base population of unselected broilers (Lassiter et al., 2025) or to low water efficient (LWE) broilers (Aloui et al., 2023).

The ability of an animal to maintain water balance depends on coordination of a wide range of hormonal systems; e.g., hypothalamic regulation of plasma osmolality, water reabsorption by the kidneys, and reabsorption of fluids in the intestines. In mammals, it is well known that the kidney is the primary organ for producing a concentrated urine and plays a major role in osmoregulation. In birds, however, the intestines play a much greater role in water reabsorption as well as water and ion balance homeostasis. When urine enters the cloaca, there is movement of the contents into the colon and cecum where water reabsorption and ion transport occurs to facilitate water and ion balance in the animal (Thomas and Skadhauge, 1979; Del la Horra et al., 1998; Braun, 1999; 2003; Laverty and Skadhauge, 1999; Nishimura and Fan, 2003; Mazancova et al., 2005).

Antidiuretic hormone (arginine vasopressin [AVP] in mammals, (arginine vasotocin [AVT] in avian species) enhances renal reabsorption of water in the collecting ducts by binding to AVP receptor 2 (AVPR2) that initiates a cAMP-mediated cascade mechanism leading to insertion of vesicles containing aquaporin 2 (AQP2) proteins into the luminal membrane. Recently, it was reported that collecting duct cells synthesize AVP, thus facilitating water reabsorption from locally produced AVP in response to increased osmolarity (Arroyo et al., 2022). Birds also have mesotocin (MT) that is structurally similar to AVT. MT causes diuresis through shunting blood from mammalian to reptilian type nephrons (Robinzon et al., 1988). A positive relationship between renal blood flow (tissue perfusion) and MT indicates MT may increase avian kidney blood flow and contribute to the diuresis mediated by MT (Bottje et al., 1989). Four AVT/MT receptors have been identified in birds; AVPR1a (Selvam et al., 2015), AVPR1b (Cornett et al., 2003), AVPR2 (Tan et al., 2000) and MTR (Gubrij et al., 2005). It is not clear whether these genes are present and proteins are functional in the intestines of birds.

Recently, it was reported that broilers with low residual water intake (water efficient), produced lower moisture of excreta and elevated mRNA expression of AVT in the hypothalamus compared to those that were water inefficient (i.e., high residual water intake phenotype) (Aggrey et al., 2023). Residual water intake is similar to residual feed intake that is used to assess feed efficiency differences in cattle that was introduced by Koch et al. (1963). The finding of elevated hypothalamic AVT expression in water efficient birds reported by Aggrey et al. (2023) is opposite to that reported in HWE broilers by Aloui et al. (2023). Also, plasma protein and mRNA expression of AVT was lower in HWE compared to LWE broilers (Greene et al., 2024). The reason for the difference in hypothalamic expression of AVT between Aggrey et al. (2023) and that reported by Aloui et al. (2023) is not apparent at this time. Renal AVT mRNA expression was elevated in HWE compared to LWE broilers which concurs with the report by Arroyo and would suggest that renal reabsorption of water would be greater in the HWE line (Lassiter et al., 2025).

As indicated above, the lower intestines in birds assume a greater role in overall water homeostasis compared to mammals. Greene et al. (2025) reported that FITC clearance was greater in LWE broilers indicating greater water excretion from the gastrointestinal tract compared to HFE broilers. Thus, we hypothesize that there will be gene expression differences that would enhance water reabsorption and reduce water loss in the gastrointestinal tract in the HWE compared to the unselected base broiler population (modern random bred, MRB). Consequently, the aim of the present study was to conduct targeted gene (mRNA) expression of the arginine vasotocin system (AVT, the antidiuretic hormone in non-mammalian animals), and the renin angiotensin system (RAS), members of the aquaporin family (AQPs), and genes that encode for tight junction related proteins (occluding [OCLN], Claudin 5 [CLDN5] and [CLDN15]) in the upper and lower duodenum and the cecum of MRB and HWE broilers maintained under thermoneutral and heat stress conditions. Differences in the expression of these different systems could help pinpoint mechanisms in the HWE broilers that contribute to increased water use efficiency.

## 2 Materials and methods

### 2.1 Ethics statement

The present study was conducted in accordance with the recommendations in the guide for the care and use of laboratory animals of the National Institutes of Health and the protocol was approved by the Division of Agriculture, University of Arkansas Animal Care and Use Committee (protocol # 23015). Tissue samples in this study were obtained previously (Lassiter et al., 2025) as an effort to reduce numbers of animals being used in these water efficiency studies. Reducing the number of animal studies used in research is an expressed desire made by federal funding agencies such as USDA.

### 2.2 Effect of chronic heat stress on MRB and HWE broilers

The MRB broiler line is a composite of four commercial broiler lines obtained from commercial genetic companies consisting of Cobb MX x Cobb500, Ross 544 x Ross 308, Ross Yield x Ross708 and Hubbard HiY line (Orlowski et al., 2020). After three generations of reciprocal mating to integrate these genetic lines, it now acts as an unselected control base population (Orlowski et al., 2020).

Tissue samples in this study were obtained on d49 from MRB and HWE male broilers from a previous study (Lassiter et al., 2025) that were maintained under thermoneutral (TN) (constant 25 °C, d28 to d49) or exposed to chronic cyclic heat stress (35 °C, 8 h/day, d28 to d49) (Lassiter et al., 2025). There were four treatments in the 2 × 2 factorial design study (2 lines x two environmental conditions). On d49, following cervical dislocation of 1 bird per pen (6 birds/treatment), ∼1 g of upper duodenum, lower duodenum and cecum (whole gut segments) was obtained, rinsed in ice cold saline, and immediately flash frozen in liquid nitrogen. Body weights (BW), feed intake (FI), and water intake (WI) were measured weekly and used to calculate feed conversion ratio (FCR) and water conversion ratio (WCR) were determined and corrected for mortality (see Table 3; Lassiter et al., 2025).

### 2.3 Total RNA extraction, reverse transcription, and quantitative real-time PCR

Total RNA extraction from the intestines was conducted using TRIzol reagent (Life Technologies, Thermo Fisher Scientific, Carlsbad, CA) according to the manufacturer’s recommendations, DNase-treated, and reverse-transcribed (Quanta Biosciences, Gaithersburg, MD). The concentration and purity of RNA was determined using a Take three microvolume plate and a Synergy HT multimode microplate reader (BioTek, Winooski, VT). The reverse-transcription (RT) products (cDNAs) were amplified by real-time quantitative PCR (7,500 real-time PCR system, Applied Biosystems, Thermo Fisher Scientific, Foster City, CA) with Power SYBR Green Master Mix (Applied Biosystems). Oligonucleotide primers specific for gene expression analysis and the 18S house keeping gene were designed using Primer Express Software (Version 3.0, Applied Biosystems, Foster City, CA) and are shown in Table 1. The quantitative PCR (qPCR) cycling conditions were 50 °C for 2 min, 95 °C for 10 min followed by 40 cycles of a two-step amplification program (95 °C for 15 s and 58 °C for 1 min). At the end of the amplification, melting curve analysis was applied using the dissociation protocol from the Sequence Detection system to exclude contamination with unspecific PCR products. Relative expressions of target genes were determined by the 2^−ΔΔCt^ method (Schmittgen and Livak, 2008).

**TABLE 1:** Oligonucleotide for quantitative reverse transcription polymerase chain reaction (qRT-PCR) primers.

Gene	Accession No^a^	Primer sequence (5’ → 3′)	Orientation	Product size, bp
ACE	NM_001167732.2	ACCCAACGAAGAAAAGAGCTATTTAT	Forward	69
		GCCGGTGCCTGAATTTCTC	Reverse	
AGT	NM_001396391.2	CAGGGTTTGCTGGGATTTGT	Forward	55
		CCCTGGAGGTGCAATTGG	Reverse	
AQP1	NM_001039453.2	GGGACATCTCCTTGCAATTGATTA	Forward	118202
		GATTGGGCCAACCCAGAAGA	Reverse	
AQP2	NM_001292072	TTTGCAGCCTCCATGATGTG	Forward	56
		AGGACAGCCCGGGTGAA	Reverse	
AQP3	XM_424500	TGCTCCTGGTCCCTGACACT	Forward	58
		CTTTTGCCTTCCCATTGCA	Reverse	
AQP4	NM_001317827	CGCTCGCAGCAGCAGTAA	Forward	59
		ATGCTACCATGATGCTCTCACACT	Reverse	
AQP5	XM_001231780.7	TGGCCATCAATTCGCTCAAC	Forward	120
		TGCCATTCCTCCTGTTGTCC	Reverse	
AQP11	XM_015280878.4	ATGCTGGTGTTTGCAGGTGG	Forward	134
		CCTAAGCATGGTGCTATCCAGT	Reverse	
AT1	NM_205157.4	GCCTTAGCATCGACCGCTAT	Forward	62
		GGTACGTCGGATTCGTGACTT	Reverse	
AT2	XM_040670971.2	GGAAACCCTCCAGATCCTCTATACA	Forward	62
		GCGGCGAGCGTAACACA	Reverse	
ATP1B1	NM_205520	CGCGTGGAATGCAAGGA	Forward	62
		CCCTGGAAGCGGTCTTTGT	Reverse	
AVT	NM_205185	TCCGGGCACACTCAGCAT	Forward	81
		ATGTAGCAGGCGGAGGACAA	Reverse	
VR1a	NM_001110438.2	CCCCTGGGTCGACTCTGAA	Forward	126
		CTCTGCACGCAGTCCTGTAG	Reverse	
VR1b	NM_001031498.2	TTTTGTCATCGTGGTGGCCT	Forward	103
		GTCGGCGGACTCGTCATC	Reverse	
VR2	NM_001031479.2	TGGTCACCATTCTTCATCGCA	Forward	76
		TGGTGAATGCTGATCCTTCAGT	Reverse	
CLDN2	NM_001277622.1	CCCAGCTGATGGCAAAGG	Forward	61
		AGGCTGATGGCACCAAAATAGT	Reverse	
CLDN15	XM_046898719.1	ATATACTCGAGGGCCCATGTT	Forward	77
		ACAGCAGCTTTGAATCCCAGA	Reverse	
HSP70	NM_001006685.2	CCGTGGAGTTCCTCAGATCG	Forward	130
		GCTAAGGCGACCCTTGTCAT	Reverse	
HSP90	NM_001109785.2	CAAACTTGGCCTGGGCATTG	Forward	78
		GGTGGCATCTCCTCGGTAAC	Reverse	
MT	XM_025150346.3	AGGAGAACTTCCTGCCAACG	Forward	110
		AAGCACACAGCCCTCACTG	Reverse	
MTR	NM_001031569.2	CCAAGATCCGGACGGTCAAA	Forward	130
		ATGAAGGGGGAGGCTTCCTG	Reverse	
OCLN	NM_205128.1	CGCAGATGTCCAGCGGTTA	Forward	59
		GTAGGCCTGGCTGCACATG	Reverse	
REN	XM_025143918.3	TGCCGGGTCTTTCCATCA	Forward	65
		GGCATTTTCCACCACTAG	Reverse	
18S	AF173612	TCCCCTCCCGTTACTTGGAT	Forward	60
		GCGCTCGTCGGCATGTA	Reverse	

Gene Abbreviations: ACE (angiotensin I converting enzyme), AGT (angiotensinogen), AQP (aquaporin), AT1 and2 (angiotensin receptor 1, 2), ATP1B1 (sodium-potassium ATPase, subunit B1), AVT (arginine vasotocin), VR1a, VR1b (AVT, receptor 1a, 1b), CLND2, 15 (claudin 2, 15), HSP70,90 (heat shock protein 70, 90), MT (mesotocin), MTR (MT, receptor), OCLN (occludin), REN (renin).

### 2.4 Statistics

The 2 × 2 factorial design data were analyzed using both Tukey’s HSD multiple comparison and Student’s t-test, and by two-way ANOVA to determine main effects of line (MRB vs. HWE), temperature condition (TN vs. HS) and their interactions using Graph Pad Prism version 9.00 for Windows (Graph Pad Software, La Jolla California, USA). Differences in mean values were determined and considered significant at *P* < 0.05. The bird was the experimental unit for gene expression data.

## 3 Results

### 3.1 Growth performance (4–7 weeks) in MRB and HWE broilers under thermoneutral and chronic heat stress temperatures

Growth performance data and intestinal tissues analyzed in the present study were obtained from a previous study (Lassiter et al., 2025). During the chronic heat stress period (4–7 weeks), chamber temperatures were elevated and relative humidity decreased in the environmental chambers resulting in increased body temperature in broilers (see Lassiter et al., 2025). There were no differences in body weight gain, feed conversion ratio (FCR), or water conversion ratio (WCR), but water intake, feed intake, and water intake to feed intake ratio were lower at P = 0.08, P = 0.06, and P = 0.06, respectively, in the HWE line compared to MRB male broilers (see Table 3, p.10; Lassiter et al., 2025). Growth performance was based on floor pen data of mixed run broilers with approximately equal numbers of males and females (p. 4, Lassiter et al., 2025).

### 3.2 HSP70 and HSP90 mRNA expression

**FIGURE 1:**
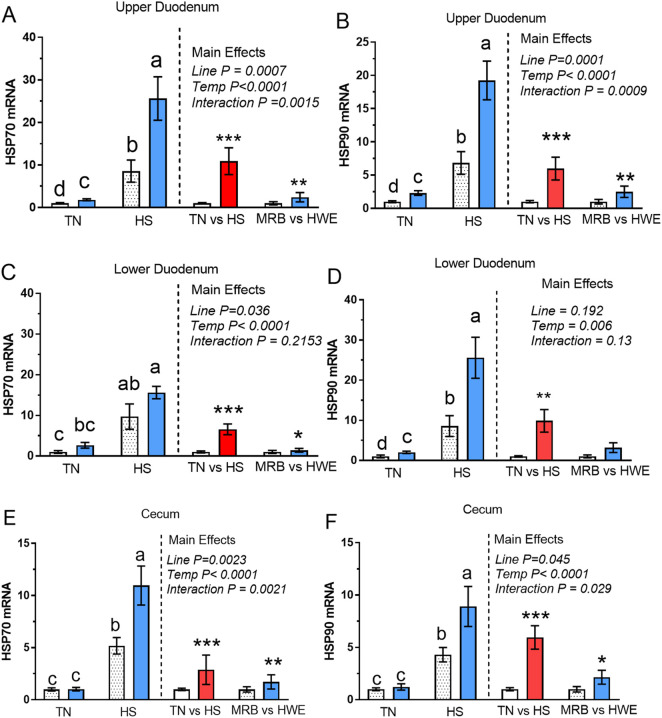
Relative gene (mRNA) expression in the intestines (upper duodenum, lower duodenum, and cecum) of heat shock protein 70 (HSP70) and heat shock protein 90 (HSP90) in modern random bred (MRB) and low water conversion ratio (HWE) male broilers maintained under thermoneutral (TN) and chronic cyclic heat stress (HS) conditions. Values for 2×2 factorial (left side of each graph) represent the mean ± SE of four to six observations for MRB (White stippled bar) and HWE (blue bar). Values for main effects (right side of each graph) represent the mean ± SE of 9 to 12 observations for the main effect of temperature (TN, white bar and HS, red bar) and broiler line (MRB, red bar) and main effect of line (MRB, white bar and HWE blue bar). ab Means with different letters are different (P < 0.05). *,**,*** Means are different at P < 0.05, P < 0.01, and P < 0.001.

The mRNA expressions of HSP70 and HSP90 in the three intestine segments are presented in Figure 1. In the upper duodenum, both HSP70 and HSP90 expressions were higher in HWE compared to values in the MRB group in both TN and HS conditions (Figures 1A,B). HS upregulated HSP70 and HSP90 in both MRB and HWE lines in the upper duodenum. Main effects were observed for both environment (HS vs. TN) and broiler line (HWE vs. MRB). A similar gene expression pattern was observed in the lower duodenum (Figures 1C,D) and for the cecum (Figures 1E,F) except there was no main effect of line for HSP90 expression in the lower duodenum (Figure 1D). There were also no differences between HSP70 and HSP90 expression in the cecum between lines under TN conditions (Figures 1E,F).

### 3.3 Intestinal mRNA expression for the AVT system, the RAS system, and the aquaporins and claudins

**FIGURE 2:**
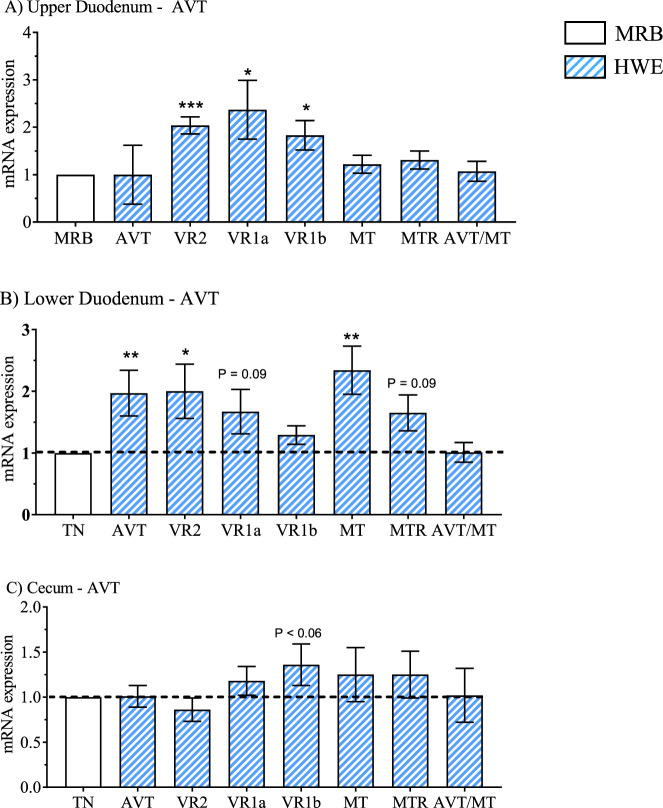
Relative mRNA expression of the arginine vasotocin (AVT) group due to broiler line (data obtained from Tables 3, 5 and 7) in the high water efficiency (HWE) broilers (blue hatched bars) compared to modern random bred (MRB) broilers (open bar). Genes include AVT, mesotocin (MT), their receptors (VR2, VR1a, VR1b, MTR) and the AVT/MT ratio in; the **(A)** Upper Duodenum, **(B)** Lower Duodenum, and **(C)** Cecum Bars represent the mean +SE (n = 9–12). *, **, *** indicates significance at P < 0.05, P < 0.01, P < 0.001 respectively.

**FIGURE 3:**
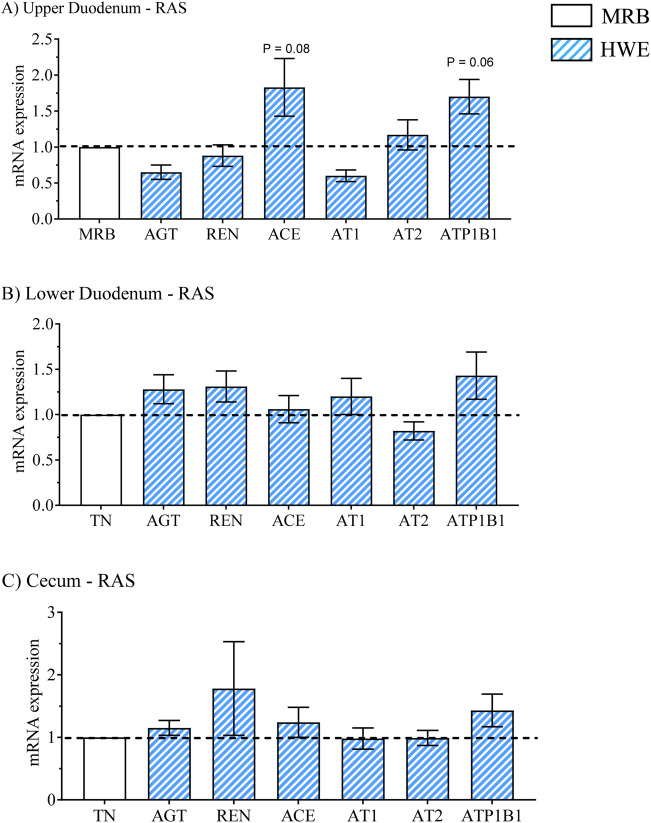
Relative mRNA expression of the renin angiotensin system (RAS) due to broiler line (data obtained from Tables 3, 5 and 7) in the high water efficiency (HWE) broilers (blue hatched bars) compared to modern random bred (MRB) broilers (open bar). Genes include angiotensinogen (AGT), renin (REN), AGT converting enzyme (ACE), angiotensin II receptors (AT1, AT2), and sodium-potassium ATPase, subunit B1 (ATP1B1) in the **(A)** Upper Duodenum, **(B)** Lower Duodenum, and **(C)** Cecum. Bars represent the mean ± SE (n = 9–12).

**FIGURE 4:**
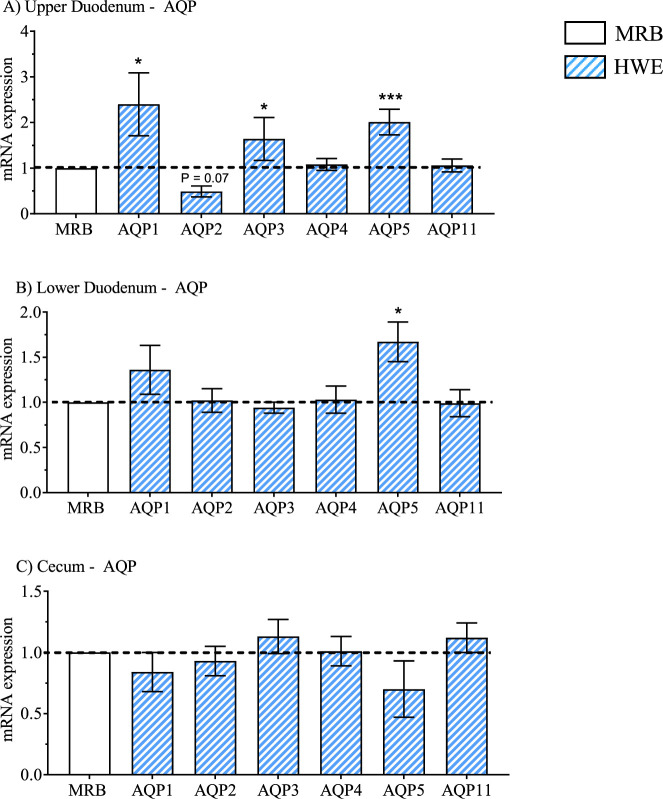
Relative mRNA expression of aquaporins (AQP) due to broiler line (data shown in Tables 3, 5 and 7) including AQP1-5 and 11 in the high water efficiency (HWE) broilers (blue hatched bars) compared to modern random bred (MRB) broilers (open bar) in the **(A)** Upper Duodenum, **(B)** Lower Duodenum, and **(C)** Cecum. Bars represent the mean ± SE (n = 9–12). *, **, *** indicates significance at P < 0.05, P < 0.01, P < 0.001 respectively.

**FIGURE 5:**
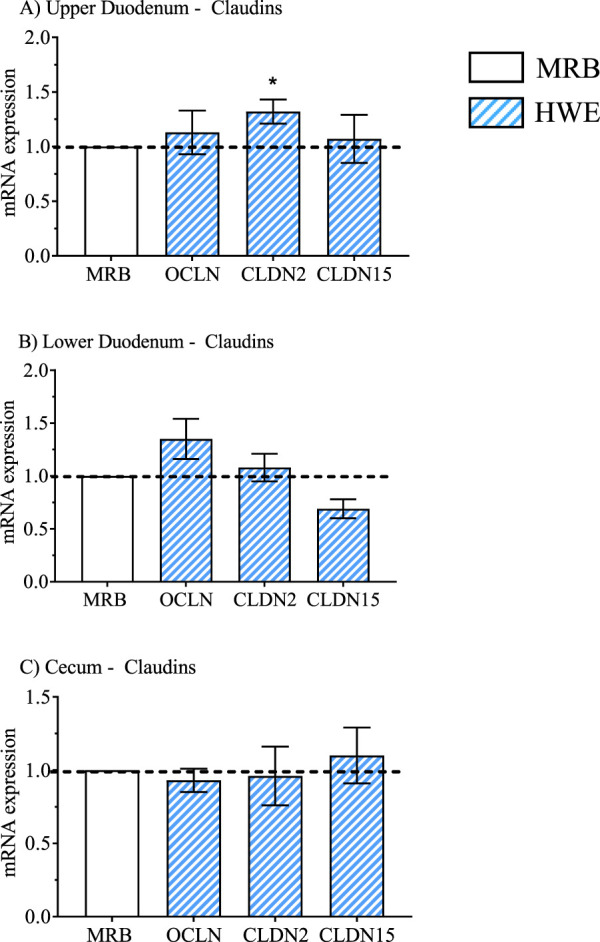
Relative mRNA expression for genes that encode expression of tight junction proteins Occludin (OCLN) and claudin two and 15 (CLDN2, 15) (data obtained from Tables 3, 5 and 7) in the high water efficiency (HWE) broilers (blue hatched bars) compared to modern random bred (MRB) broilers (open bar) in the **(A)** Upper Duodenum, **(B)** Lower Duodenum, and **(C)** Cecum. Bars represent the mean ± SE (n = 9–12). *, **, *** indicates significance at P < 0.05, P < 0.01, P < 0.001 respectively.

**FIGURE 6:**
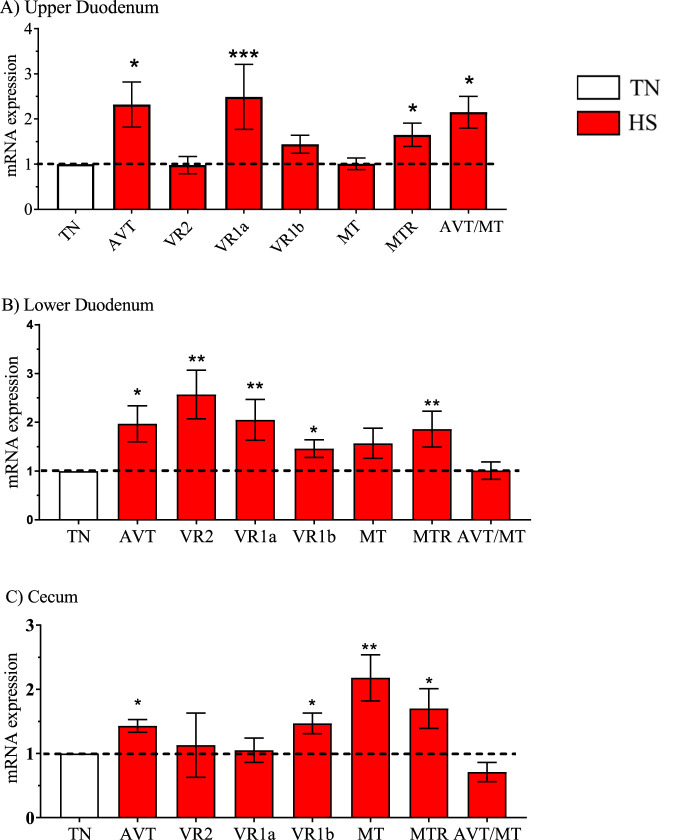
Relative mRNA expression of the arginine vasotocin (AVT) group due to a main effect of heat stress (HS) (red bars) (data obtained from Tables 3, 5 and 7) compared to broilers maintained in a thermoneutral (TN) environment (open bar). Genes include AVT, mesotocin (MT), their receptors (VR2, VR1a, VR1b, MTR) and the AVT/MT in the **(A)** Upper Duodenum, **(B)** Lower Duodenum, and **(C)** Cecum Bars represent the mean ± SE (n = 9) *, **, *** indicates significance at P < 0.05, P < 0.01, P < 0.001 respectively.

**FIGURE 7:**
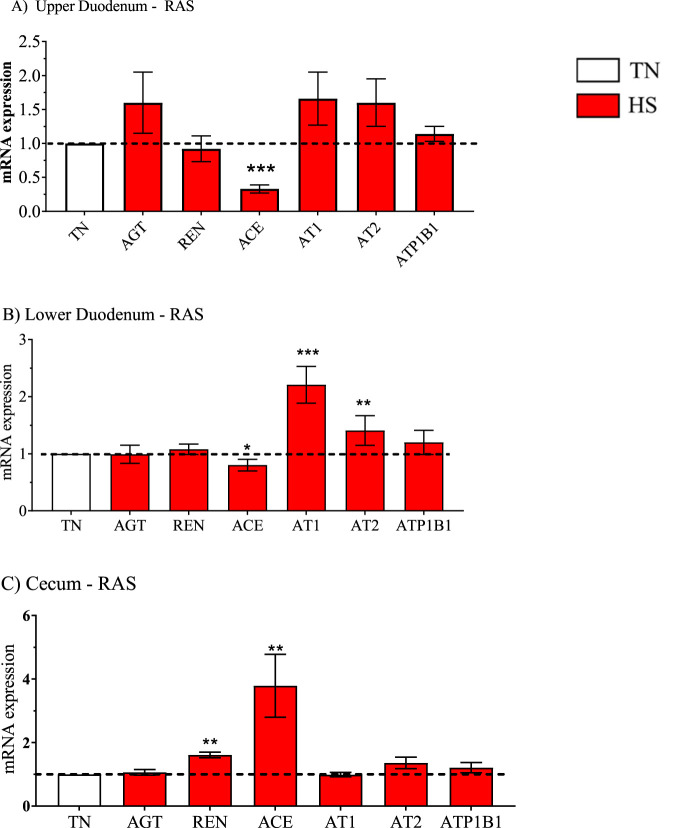
Relative mRNA expression of the renin angiotensin system (RAS) in heat-stressed broilers (red bars) (data obtained from Tables 3, 5 and 7) compared to broilers maintained in a thermoneutral (TN) environment (open bar). Genes include angiotensinogen (AGT), renin (REN), AGT converting enzyme (ACE), angiotensin II receptors (AT1, AT2), and sodium-potassium ATPase, subunit B1 (ATP1B1) in the **(A)** Upper Duodenum, **(B)** Lower Duodenum, and **(C)** Cecum Bars represent the mean ± SE (n = 9–12). *, **, *** indicates significance at P < 0.05, P < 0.01, P < 0.001 respectively.

**FIGURE 8:**
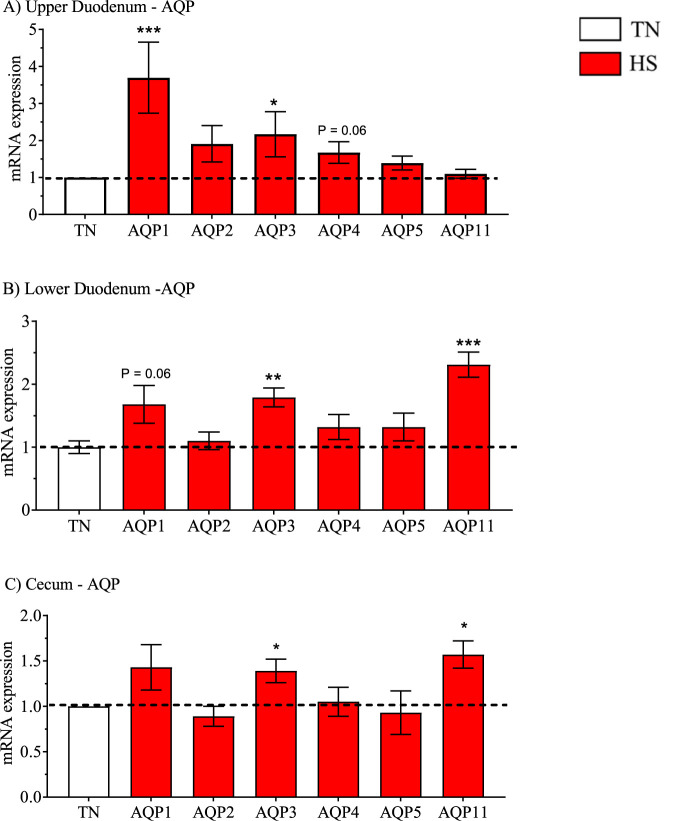
Relative mRNA expression of aquaporins (AQP) including AQP1-5 and 11 in heat-stressed broilers (red bars) (data obtained from Tables 3, 5 and 7) compared to broilers maintained in a thermoneutral (TN) environment (open bars) in the **(A)** Upper Duodenum, **(B)** Lower Duodenum, and **(C)** Cecum. Bars represent the mean ± SE (n = 9–12). *, **, *** indicates significance at P < 0.05, P < 0.01, P < 0.001 respectively.

**FIGURE 9:**
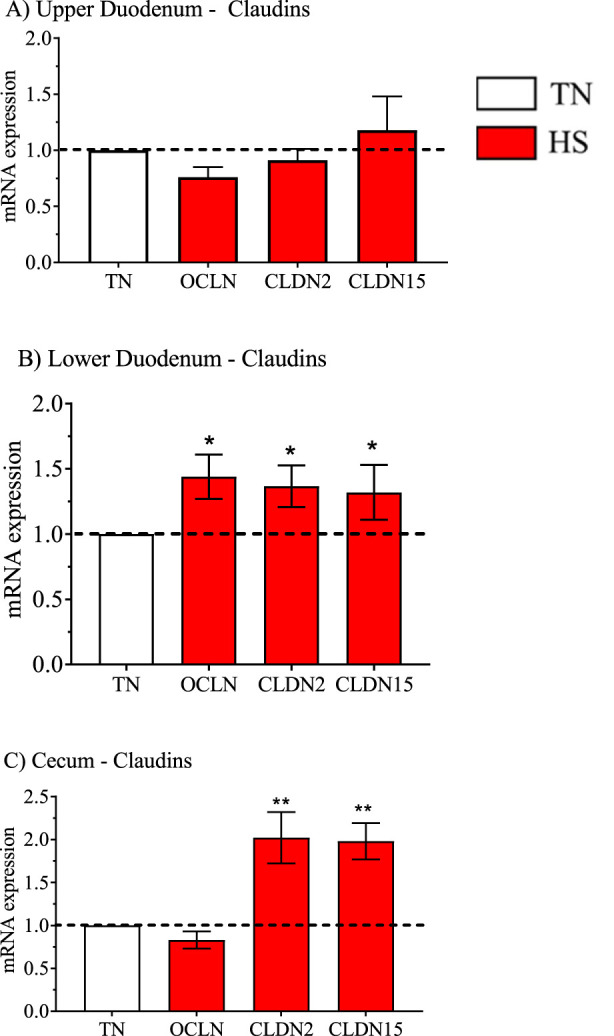
Relative mRNA expression of genes that encode expression of Occludin (OCLN) and claudin two and 15 (CLDN2, 15) in heat stressed broilers (data obtained from Tables 3, 5 and 7) compared to in the high water efficiency (HWE) broilers (hatched bars) compared to broilers maintained in a thermoneutral (TN) environment (open bars) in the **(A)** Upper Duodenum, **(B)** Lower Duodenum, and **(C)** Cecum. Bars represent the mean ± SE (n = 9–12). *, **, *** indicates significance at P < 0.05, P < 0.01, P < 0.001 respectively.

The mRNA expression results for the 2 × 2 factorial design for the upper duodenum, lower duodenum, and cecum are presented in Tables 2, 4, 5 whereas main effects of broiler line (MRB vs. HWE) and environment (TN vs. HS) are presented in Tables 3, 6, and 7. Main effects on gene expression between HWE to MRB (standardized to a common MRB value of 1.0) are presented in Figures 2–5 and main effects due to environment (TN vs. HS) standardized to a common value under TN conditions of 1.0 are presented in Figures 6–9.

**TABLE 2:** Upper duodenum relative mRNA expression the Arginine Vasotocin (AVT) system, Renin-Angiotensin System (RAS), Aquaporin (AQP) system, and genes encoding for claudins (tight junction proteins) in modern random bred (MRB) and high water efficient (HWE) broiler lines under thermoneutral (TN) and chronic cyclic heat stress (HS) environments (2 × 2 factorial, two way ANOVA)^a^.

Target system/Gene^b^	MRB-TN			HWE-TN			MRB-HS			HWE-HS			2 way ANOVA		
Upper duodenum	Mean	SE		Mean	SE		Mean	SE		Mean	SE		Line (L)	Env (E)	LxE
AVT system															
AVT	1.00	0.26	b	1.06	0.25	bc	2.44	0.17	a	2.35	0.49	a	ns	0.0003	ns
VR2	1.00	0.23	b	1.97	0.17	a	1.01	0.15	b	2.20	0.36	a	0.0002	ns	ns
VR1a	1.00	0.15	c	1.78	0.52	bc	2.02	0.45	ab	5.48	1.53	a	0.0204	0.0110	ns
VR1b	1.00	0.14	b	2.19	0.54	ab	1.56	0.33	ab	2.41	0.54	a	0.0260	ns	ns
MT	1.00	0.16	a	1.57	0.32	a	1.35	0.15	a	1.25	0.29	a	ns	ns	ns
MTR	1.00	0.12	b	1.77	0.29	a	2.23	0.56	a	2.36	0.50	a	ns	0.0280	ns
AVT/MT	1.00	0.14	cb	0.75	0.11	c	1.63	0.56	ab	2.13	0.33	a	ns	0.0045	ns
RAS system															
AGT	1.00	0.35	a	1.01	0.25	a	2.15	0.79	a	1.06	0.20	a	ns	ns	ns
REN	1.00	0.21	a	1.28	0.24	a	1.33	0.28	a	0.72	0.18	a	ns	ns	0.068
ACE	1.00	0.18	a	1.14	0.12	a	0.48	0.10	b	0.25	0.02	b	ns	<0.0001	ns
AT1	1.00	0.34	a	1.06	0.22	a	2.17	0.69	a	1.35	0.23	a	ns	0.0638	ns
AT2	1.00	0.14	b	1.60	0.20	a	2.14	0.65	ab	2.11	0.65	ab	ns	0.0885	ns
ATP1B1	1.00	0.25	a	1.50	0.32	a	0.77	0.14	a	1.20	0.17	a	0.0578	ns	ns
Aquaporin system															
AQP1	1.00	0.34	c	2.74	0.64	b	3.4	0.62	b	11.31	2.26	a	0.0001	<0.0001	0.0062
AQP2	1.00	0.44	a	0.67	0.15	a	1.95	0.68	a	1.24	0.39	a	ns	ns	ns
AQP3	1.00	0.23	b	1.00	0.28	b	1.7	0.53	ab	3.33	0.96	a	ns	0.0109	ns
AQP4	1.00	0.16	b	1.35	0.23	b	1.2	0.26	ab	2.71	0.46	a	0.0038	0.0122	0.054
AQP5	1.00	0.16	bc	1.63	0.33	b	0.73	0.08	c	1.93	0.35	a	0.0024	ns	ns
AQP11	1.00	0.17	a	1.11	0.27	a	1.19	0.19	a	1.22	0.16	a	ns	ns	ns
Tight junction															
OCLN	1.00	0.25	a	1.44	0.33	a	0.97	0.14	a	0.84	0.15	a	ns	ns	ns
CLDN2	1.00	0.27	a	1.48	0.15	a	1.09	0.20	a	1.18	0.13	a	ns	ns	Ns
CLDN15	1.00	0.18	b	3.15	0.45	a	3.97	0.81	a	1.81	0.70	ab	ns	ns	0.0036

^a^ Values represent the mean and standard error (SE) of four to six observations.

^b^ See footnote Table 1 for abbreviations.

abc Means with different letters are different (P < 0.05); ns indicates no significant difference in main effects.

**TABLE 3:** Main effects of broiler line (modern random bred, MRB, and high water efficiency, HWE) and environment (thermoneutral, TN and cyclic heat stress, HS), on mRNA expression in the upper duodenum for the Arginine Vasotocin (AVT) system, Renin-Angiotensin System (RAS), Aquaporin (AQP) system, and genes encoding for claudins (tight junction proteins)^a^.

Target system/Gene^b^	Main effect: Broiler line							Main effect: Environment						
Upper duodenum	MRB			HWE				TN			HS			
	Mean	SE	n	Mean	SE	n	P =	Mean	SE	N	Mean	SE	n	P =
AVT system														
AVT	1.00	0.30	11	1.00	0.62	11	ns	1.00	0.17	11	2.32	0.50	11	0.013
VR2	1.00	0.14	11	2.04	0.18	9	0.0001	1.00	0.14	12	0.98	0.19	9	ns
VR1a	1.00	0.19	11	2.37	0.62	11	0.042	1.00	0.20	12	2.49	0.72	10	<0.0001
VR1b	1.00	0.15	11	1.83	0.31	10	0.030	1.00	0.20	11	1.44	0.20	10	ns
MT	1.00	0.11	11	1.22	0.19	12	ns	1.00	0.15	11	1.01	0.13	11	ns
MTR	1.00	0.21	11	1.31	0.19	11	ns	1.00	0.14	12	1.65	0.26	10	0.044
AVT/MT	1.00	0.19	10	1.07	0.21	11	ns	1.00	0.13	12	2.15	0.35	10	0.002
RAS system														
AGT	1.00	0.29	10	0.65	0.10	11	ns	1.00	0.2	11	1.6	0.45	10	ns
REN	1.00	0.15	12	0.88	0.15	11	ns	1.00	0.14	11	0.92	0.19	12	ns
ACE	1.00	0.21	12	1.83	0.40	12	0.075	1.00	0.10	12	0.33	0.06	12	<0.0001
AT1	1.00	0.31	10	0.6	0.08	11	ns	1.00	0.19	10	1.66	0.39	9	0.100
AT2	1.00	0.26	11	1.17	0.21	11	ns	1.00	0.12	11	1.6	0.35	10	0.09
ATP1B1	1.00	0.12	12	1.7	0.24	12	0.060	1.00	0.17	12	1.14	0.11	12	ns
Aquaporin system														
AQP1	1.00	0.25	12	2.40	0.69	10	0.020	1.00	0.24	12	3.70	0.96	10	0.004
AQP2	1.00	0.25	12	0.49	0.12	12	0.070	1.00	0.27	12	1.91	0.49	12	ns
AQP3	1.00	0.22	11	1.64	0.47	12	ns	1.00	0.18	12	2.17	0.61	11	0.020
AQP4	1.00	0.18	12	1.08	0.13	11	ns	1.00	0.14	12	1.67	0.29	11	0.060
AQP5	1.00	0.12	11	2.01	0.28	10	0.0003	1.00	0.17	12	1.39	0.19	10	ns
AQP11	1.00	0.12	12	1.06	0.14	12	ns	1.00	0.17	12	1.1	0.12	12	ns
Tight junction														
OCLN	1.00	0.15	12	1.13	0.20	11	ns	1.00	0.17	11	0.76	0.09	12	ns
CLDN2	1.00	0.12	12	1.32	0.11	12	0.041	1.00	0.14	12	0.91	0.10	12	ns
CLDN15	1.00	0.30	8	1.07	0.22	11	ns	1.00	0.21	10	1.18	0.30	10	ns

^a^ Values represent the mean +SE, of 8–12 observation and probability (P) values of significance or not significant (ns) as indicated.

^b^ See footnote Table 1 for abbreviations.

**TABLE 4:** Lower duodenum relative mRNA expression of the Arginine Vasotocin (AVT) system, Renin-Angiotensin System (RAS), Aquaporin (AQP) system, and genes encoding for claudins (tight junction proteins) in modern random bred (MRB) and high water efficient (HWE) broiler lines under thermoneutral (TN) and chronic cyclic heat stress (HS) environments (2 × 2 factorial, two way ANOVA)^a^.

Target system/Gene^b^	MRB-TN			HWE-TN			MRB-HS			HWE-HS			2 way ANOVA		
Lower duodenum	Mean	SE		Mean	SE		Mean	SE		Mean	SE		Line (L)	Temp (E)	LxE
AVT System															
AVT	1.00	0.14	b	1.18	0.16	b	2.23	0.57	a	3.12	0.50	a	ns	0.0007	ns
VR2*	1.00	0.11	b	1.30	0.26	bc	1.74	0.38	ab	4.17	0.75	a	0.0059	0.0006	0.0262
VR1a	1.00	0.14	c	1.39	0.23	bc	1.93	0.42	ab	3.52	0.077	a	0.0008	<0.0001	0.0275
VR1b	1.00	0.19	b	1.58	0.26	ab	1.91	0.33	a	2.18	0.30	a	ns	0.0125	ns
MT	1.00	0.21	b	2.84	0.77	ab	1.99	0.64	ab	4.31	0.73	a	ns	0.0758	ns
MTR	1.00	0.20	b	1.57	0.39	b	2.05	0.61	ab	3.47	0.56	a	0.0384	0.0039	ns
AVT/MT	1.00	0.44	a	0.77	0.39	a	1.08	0.23	a	0.69	0.22	a	ns	ns	ns
RAS system															
AGT	1.00	0.23	a	1.53	0.31	a	1.46	0.37	a	1.56	0.24	a	ns	ns	ns
REN	1.00	0.22	a	1.68	0.37	a	1.54	0.14	a	1.64	0.2	a	ns	ns	ns
ACE	1.00	0.16	a	1.14	0.19	a	0.68	0.11	ab	0.61	0.19	A	ns	0.0182	ns
AT1	1.00	0.14	b	1.40	0.08	b	3.10	0.53	a	3.57	0.51	a	ns	<0.0001	ns
AT2	1.00	0.24	b	1.22	0.11	ab	2.27	0.49	a	1.82	0.24	ab	ns	0.0082	ns
ATP1B1	1.00	0.13	b	0.98	0.26	ab	0.78	0.14	ab	1.6	0.32	A	0.0869	ns	ns
Aquaporin															
AQP1	1.00	0.26	b	1.56	0.36	ab	1.89	0.43	ab	2.37	0.62	ab	ns	0.0616	ns
AQP2	1.00	0.28	a	1.42	0.28	a	1.34	0.30	a	1.24	0.16	a	ns	ns	ns
AQP3	1.00	0.08	b	1.51	0.26	ab	2.09	0.32	ab	2.33	0.14	a	0.0983	0.0004	ns
AQP4	1.00	0.14	a	1.15	0.23	a	1.21	0.3	a	1.62	0.29	a	ns	ns	ns
AQP5	1.00	0.13	b	1.65	0.29	ab	1.30	0.29	ab	2.19	0.44	a	ns	ns	ns
AQP11	1.00	0.24	b	1.00	0.16	b	2.35	0.33	a	2.28	0.24	a	ns	<0.0001	ns
Tight junction															
OCLN	1.00	0.15	b	1.32	0.27	ab	1.47	0.16	ab	2.02	0.32	a	0.0806	0.0224	ns
CLDN2	1.00	0.27	b	1.12	0.23	ab	1.73	0.23	ab	1.71	0.19	a	ns	0.0114	ns
CLDN15	1.00	0.09	b	1.17	0.1	b	2.27	0.15	a	1.08	0.14	ab	0.0008	0.0002	<0.0001

^a^ Values represent the mean and standard error (SE) of four to six observations.

^b^ See footnote Table 1for abbreviations.

abc Means with different letters are different (P < 0.05); ns indicates no significant difference in main effects.

**TABLE 5:** Main effects of broiler line (modern random bred, MRB, and high water efficiency, HWE) and environment (thermoneutral, TN and cyclic heat stress, HS), on mRNA expression in the lower duodenum for the Arginine Vasotocin (AVT) system, Renin-Angiotensin System (RAS), Aquaporin (AQP) system, and genes encoding for claudins (tight junction proteins)^a^.

Target system/ Gene^b^	Main effect: Broiler line							Main effect: Environment						
Lower duodenum	MRB			HWE				TN			HS			
	Mean	SE	n	Mean	SE	N	P Value	Mean	SE	n	Mean	SE	n	P Value
AVT system														
AVT	1.00	0.10	12	1.33	0.25	12	0.31	1.00	0.10	12	2.46	0.37	12	0.001
VR2*	1.00	0.17	12	2.00	0.44	12	0.038	1.00	0.13	12	2.57	0.50	12	0.004
VR1a	1.00	0.18	12	1.67	0.36	12	0.0909	1.00	0.12	12	2.05	0.42	12	0.006
VR1b	1.00	0.16	12	1.29	0.15	12	ns	1.00	0.14	12	1.46	0.18	12	0.010
MT	1.00	0.25	10	2.34	0.39	10	0.008	1.00	0.26	10	1.57	0.31	10	ns
MTR	1.00	0.22	11	1.65	0.29	10	0.091	1.00	0.17	11	1.86	0.37	12	0.006
AVT/MT	1.00	0.27	9	1.01	0.16	8	ns	1.00	0.26	9	1.01	0.18	8	ns
RAS system														
AGT	1.00	0.19	9	1.28	0.16	10	ns	1.00	0.17	10	0.99	0.16	9	ns
REN	1.00	0.13	10	1.31	0.17	12	ns	1.00	0.19	11	1.08	0.09	11	0.414
ACE	1.00	0.14	12	1.06	0.15	11	ns	1.00	0.12	11	0.81	0.10	12	0.015
AT1	1.00	0.21	10	1.20	0.20	9	ns	1.00	0.10	10	2.21	0.32	9	0.0004
AT2	1.00	0.21	11	0.82	0.11	11	ns	1.00	0.13	12	1.41	0.26	11	0.0064
ATP1B1	1.00	0.11	11	1.43	0.26	11	0.110	1.00	0.13	11	1.20	0.21	10	ns
AQP family														
AQP1	1.00	0.2	12	1.36	0.27	10	ns	1.00	0.19	11	1.68	0.30	10	0.055
AQP2	1.00	0.16	12	1.02	0.13	12	ns	1.00	0.17	12	1.1	0.14	12	ns
AQP3	1.00	0.09	11	0.94	0.06	9	ns	1.00	0.13	12	1.79	0.15	11	0.001
AQP4	1.00	0.17	12	1.03	0.15	12	ns	1.00	0.13	12	1.32	0.20	12	ns
AQP5	1.00	0.14	12	1.67	0.22	10	0.017	1.00	0.14	11	1.32	0.22	11	ns
AQP11	1.00	0.18	12	0.99	0.15	12	ns	1.00	0.14	12	2.31	0.20	12	<0.0001
Tight junction														
OCLN	1.00	0.11	12	1.35	0.19	12	0.105	1.00	0.15	12	1.44	0.17	12	0.02
CLDN2	1.00	0.17	11	1.08	0.13	11	ns	1.00	0.17	11	1.37	0.16	12	0.01
CLDN15	1.00	0.18	12	0.69	0.09	10	ns	1.00	0.14	12	1.32	0.27	10	0.04

^a^ Values represent the mean +SE, of 8–12 observation and probability (P) values of significance or not significant (ns) as indicated.

^b^ See footnote Table 1 for abbreviations.

**TABLE 6:** Cecum relative mRNA expression of the Arginine Vasotocin (AVT) system, Renin-Angiotensin System (RAS), Aquaporin (AQP) system, and genes encoding for claudins (tight junction proteins) in modern random bred (MRB) and high water efficient (HWE) broiler lines under thermoneutral (TN) and chronic cyclic heat stress (HS) environments (2 × 2 factorial, two way ANOVA)^a^.

Cecum	MRB-TN			HWE-TN			MRB-HS			HWE-HS			2 way ANOVA		
Target system/Gene	Mean	SE		Mean	SE		Mean	SE		Mean	SE		Line (L)	Temp (E)	LxE
AVT system															
AVT	1.00	0.18	ab	0.97	0.20	b	1.31	0.12	ab	1.53	0.12	a	ns	0.0153	ns
VR2*	1.00	0.18	a	0.65	0.10	a	0.87	0.15	a	0.99	0.21	a	ns	ns	ns
VR1a	1.00	0.24	a	0.75	0.14	a	0.75	0.09	a	1.22	0.28	a	ns	ns	0.102
VR1b	1.00	0.18	b	1.28	0.13	ab	1.38	0.11	ab	1.95	0.29	a	0.0375	0.0123	ns
MT	1.00	0.23	b	0.82	0.15	ab	1.46	0.34	ab	2.60	0.56	a	ns	0.0027	0.0528
AVT/MT	1.00	0.27	a	1.07	0.33	a	0.83	0.21	a	0.47	0.16	a	ns	ns	ns
MTR	1.00	0.12	a	0.99	0.28	a	1.38	0.37	a	1.99	0.46	a	ns	0.0509	ns
RAS system															
AGT	1.00	0.17	a	1.23	0.35	a	1.38	0.12	a	0.98	0.08	a	ns	ns	ns
REN	1.00	0.10	b	1.21	0.21	b	1.70	0.13	a	1.85	0.14	a	ns	0.0002	ns
ACE	1.00	0.18	a	0.58	0.12	a	1.34	0.11	a	4.64	0.76	a	0.0026	<0.0001	0.0003
AT1	1.00	0.17	a	1.72	0.51	a	1.46	0.14	a	1.23	0.11	a	ns	ns	ns
AT2	1.00	0.13	a	1.34	0.38	a	1.79	0.30	a	1.40	0.27	a	ns	ns	ns
ATP1B1	1.00	0.15	a	0.84	0.12	a	1.02	0.19	a	1.23	0.20	a	ns	ns	ns
Aquaporin															
AQP1	1.00	0.15	a	0.65	0.22	a	1.16	0.34	a	1.21	0.20	a	ns	ns	ns
AQP2	1.00	0.17	a	0.93	0.17	a	0.90	0.11	a	0.83	0.19	a	ns	ns	ns
AQP3	1.00	0.14	a	1.12	0.13	a	1.36	0.15	a	1.55	0.21	a	ns	0.0256	ns
AQP4	1.00	0.16	a	0.71	0.14	a	0.86	0.12	a	0.94	0.22	a	ns	ns	ns
AQP5	1.00	0.18	a	0.59	0.11	a	0.71	0.24	a	0.81	0.34	a	ns	ns	ns
AQP11	1.00	0.22	b	1.12	0.24	b	1.41	0.17	b	1.97	0.18	a	ns	0.0069	ns
Tight junction															
OCLN	1.00	0.12	a	0.83	0.10	a	0.92	0.14	a	0.99	0.12	a	ns	ns	ns
CLDN2	1.00	0.18	ab	0.66	0.20	b	1.62	0.35	a	1.80	0.37	a	ns	0.006	ns
CLDN15	1.00	0.44	ab	1.02	0.22	b	2.02	0.32	a	1.98	0.51	ab	ns	0.0159	ns

^a^ Values represent the mean and standard error (SE) of four to six observations.

^b^ See footnote Table 1 for abbreviations. abc Means with different letters are different (P < 0.05); ns indicates no significant difference in main effects.

**TABLE 7:** Main effects of broiler line (modern random bred, MRB, and high water efficiency, HWE) and environment (thermoneutral, TN and cyclic heat stress, HS), on mRNA expression in the cecum for the Arginine Vasotocin (AVT) system, Renin-Angiotensin System (RAS), Aquaporin (AQP) system, and genes encoding for claudins (tight junction proteins)^a^.

Target system/ Gene^b^	Main effect: Broiler line							Main effect: Environment						
Cecum	MRB			HWE				TN			HS			
	Mean	SE	n	Mean	SE	n	P Value	Mean	SE	N	Mean	SE	n	P Value
AVT system														
AVT	1.00	0.10	11	1.01	0.12	12	ns	1.00	0.13	12	1.43	0.10	12	0.013
VR2*	1.00	0.12	11	0.86	0.13	11	ns	1.00	0.14	12	1.13	0.15	11	ns
VR1a	1.00	0.15	11	1.18	0.16	10	ns	1.00	0.16	11	1.05	0.19	10	ns
VR1b	1.00	0.10	12	1.36	0.23	12	0.056	1.00	0.10	12	1.47	0.16	12	0.038
MT	1.00	0.18	10	1.25	0.30	10	ns	1.00	0.15	11	2.18	0.36	9	0.007
MTR	1.00	0.17	12	1.25	0.26	12	ns	1.00	0.15	12	1.70	0.31	12	0.047
AVT/MT	1.00	0.18	10	1.02	0.30	8	ns	1.00	0.20	11	0.71	0.15	9	ns
RAS system														
AGT	1.00	0.19	9	1.28	0.16	10	ns	1.00	0.17	10	0.99	0.16	9	ns
REN	1.00	0.14	12	1.15	0.12	12	ns	1.00	0.11	12	1.61	0.09	12	0.0001
ACE	1.00	0.14	10	1.78	0.75	10	ns	1.00	0.17	10	3.79	0.99	9	0.010
AT1	1.00	0.11	12	1.24	0.24	11	ns	1.00	0.11	12	0.99	0.07	10	ns
AT2	1.00	0.15	12	0.98	0.17	12	ns	1.00	0.17	12	1.36	0.18	12	ns
ATP1B1	1.00	0.13	11	0.99	0.12	11	ns	1.00	0.11	11	1.21	0.16	11	ns
Aquaporin family														
AQP1	1.00	0.17	11	0.84	0.16	11	ns	1.00	0.17	11	1.43	0.25	10	ns
AQP2	1.00	0.11	11	0.93	0.12	11	ns	1.00	0.12	12	0.89	0.11	11	ns
AQP3	1.00	0.10	10	1.13	0.14	10	ns	1.00	0.08	10	1.39	0.13	10	0.021
AQP4	1.00	0.16	12	1.01	0.12	12	ns	1.00	0.13	12	1.05	0.16	12	ns
AQP5	1.00	0.16	11	0.70	0.23	10	ns	1.00	0.18	11	0.93	0.24	11	ns
AQP11	1.00	0.13	12	1.12	0.12	11	ns	1.00	0.16	12	1.57	0.15	11	0.011
Tight junction														
OCLN	1.00	0.10	12	0.93	0.08	10	ns	1.00	0.12	12	0.83	0.10	12	ns
CLDN2	1.00	0.16	11	0.96	0.20	10	ns	1.00	0.17	11	2.02	0.30	10	0.005
CLDN15	1.00	0.21	12	1.10	0.19	11	ns	1.00	0.23	12	1.98	0.21	11	0.010

^a^ Values represent the mean +SE, of the number (n) of observations and probability (P) values of significance or not significant (ns) as indicated.

^b^ See footnote Table 1 for abbreviations.

#### 3.3.1 Gene expression for 2 × 2 factorial

Under TN conditions, genes that were upregulated in the AVT system in the upper duodenum of HWE broilers included VR2, MTR, AT2, AQP1, and CLDN15 (Table 2). In the lower duodenum, only VR1b, MT, and AT1 were upregulated in the HWE group (Table 4) and there were no differences in gene expression in the cecum between MRB and HWE groups under TN conditions (Table 5). Under HS conditions, HWE birds had higher expression of VR2, AQP1, and AQP5 in the upper duodenum than in the MRB group but there were no differences between groups due to HS in the lower duodenum or cecum.

#### 3.3.2 Main effect of broiler line

Gene expression in HWE compared to MRB broilers in the upper duodenum is presented in Table 3 and in Figures 2–5 for the AVT group, the RAS system, the AQP family, and three claudins (that encode for tight junction proteins), respectively. The expression of VR2, VR1a and VR1b mRNA was elevated in HWE in the upper duodenum (Figure 2A). AVT, VR2, and MT were also elevated in the lower duodenum, and VR1b elevated (P < 0.06) in the cecum (Figure 2C) in HWE birds. There were no differences in mRNA expression of RAS system genes in each gut segment with the exception of ATP1B1 (P = 0.06) in the upper duodenum (Figure 3A). Expression of AQP genes is presented in Figure 4. AQP1, three and five expressions were higher and AQP2 was lower (P = 0.07) in the HWE group compared to the MRB group in the upper duodenum (Figure 4A). AQP5 expression was higher in the HWE group (Figure 4B) but there were no differences in AQP expression in the cecum (Figure 4C). Only CLDN2 was elevated in the HWE group (Figure 5A), but there were no differences between groups in expression in the claudin family in the lower duodenum (Figure 5B) or cecum (Figure 5C).

#### 3.3.3 Main effect of environment

HS resulted in elevated mRNA expression of several members of the AVT family in each gut segment compared to that obtained from TN maintained birds. (Figure 6). AVT expression was elevated in all three gut segments. In addition, VR1a, MTR and AVT/MT were elevated in the upper duodenum (Figure 6A), while VR2, VR1a, VR1b and MTR were elevated in the lower duodenum (Figure 6B), and VR1b, MT, and MTR were elevated in the cecum (Figure 6C). None of the AVT family genes were downregulated in HWE broilers.

For the RAS system of genes presented in Figure 7, ACE was downregulated in the upper and lower duodenum but was elevated in the cecum. Both AT1 and AT2 expression was upregulated in HS in the lower duodenum (Figure 7B) whereas REN was upregulated by HS in the cecum (Figure 7C).

With respect to water transport related genes, AQP1, AQP3, and AQP4 (P = 0.06) were upregulated in the upper duodenum in response to HS (Figure 8A). HS also increased mRNA expression of AQP1 (P = 0.06), AQP3 and AQP11 in the lower duodenum (Figure 8B) as well as AQP3 and AQP11 in the Cecum (Figure 8C). HS had no effect on any claudin-related genes in the upper duodenum (Figure 9A), but increased expression of OCLN, CLDN2 and CLDN15 in the lower duodenum (Figure 9B) and CLDN2 and CLDN15 in the cecum (Figure 9C).

## 4 Discussion

Intestinal samples in this study were obtained previously and genes targeted were the same as those reported in the kidney (see Exp. 2, Lassiter et al., 2025). This was done intentionally in an effort to gain a more complete global understanding of the impact of genetic selection for water efficiency in these unique broiler lines.

In mammals, the gastrointestinal tract is responsible for bulk water transport, and as an organ, is second only to the kidney in movement of fluids (Ma and Verkman, 1999; Laforenza, 2012). In humans, ∼2L of water enters the gut per day through drinking and ∼7L by gastrointestinal secretions with only 100 mL of water being excreted in feces. Water movement across gut epithelial cells is bidirectional via reabsorption and secretion processes and is regulated by a wide range of neurotransmitters, gastrointestinal hormones, inflammatory factors and by content within the gut lumen. The results of this study will be discussed in five groups of genes for the upper duodenum, lower duodenum, and cecum focusing on; HSPs (4.1), the AVT system (4.2), the RAS system (4.3), members of the Aquaporin (AQP) family (4.4), and genes of the claudin family that encode tight junction proteins (4.5). These were the same genes that were examined in the kidney reported previously (Lassiter et al., 2025).

### 4.1 Heat shock proteins

The first report of heat shock protein response is credited to Ritossa (1962) who observed ‘puffing’ of chromosomes by microscopy in fruit flies in response to temperature shock. This was followed by similar reports in different organisms and extended to include stress response to endotoxins, hypoxia, ischemia, and oxidative stress (Ritossa, 1964). Siddiqui et al. (2020) reported that elevated HSP60 and HSP70 in intestines served to activate adaptive immunity enabling broilers to adapt to chronic heat stress conditions. Acute heat stress resulted in a biphasic change in HSP60, HSP70 and HSP47 with expression of both mRNA and proteins increasing from 0 to 6 h and then returning to control values by 24 h (Siddiqui et al., 2020b). In the kidney, HSP90 was higher (P = 0.05) in HWE broilers (Lassiter et al., 2025).

Boopathy et al. (2022) reviewed the role that HSPs play in proteostasis (protein homeostasis) and challenges such as heat stress, toxic shock, and reactive oxygen species. HSPs also provide scaffolding and chaperone functions to maintain cell proteins in their optimal tertiary conformation (Hu et al., 2022). Thus, not only are HSPs crucial for preserving molecular function during stress but are also essential for maintaining normal cell function under normal conditions.

The HWE line exhibited higher expression of both HSP70 and HSP90 in all three gut segments compared to MRB expression (main effect of line) with the exception of HSP90 in the lower duodenum (Figure 1). A main effect of HS resulted in higher HSP70 and HSP90 expression in all three gi segments. The expression of HSP70 and HSP90 were also higher in HWE compared to MRB in each gut segment with the exception of HSP70 in the lower duodenum (Figure 1). From the discussion above, it appears that HWE birds would likely have greater capability for maintaining tertiary structures and in reducing damage from oxidative stress, and thus maintain protein functionality), compared to the MRB. With the dynamic nature of the mucosal layer of the gut with rapid cell turnover that is mediated in part by mitochondrial ROS production, it is possible that functionality of cells might be better maintained in HWE birds. There is indication that HSPs can stabilize tight junction proteins and aquaporin channels that could be hypothesized to have a direct impact on water movement in the intestinal tract (Wang et al., 2017).

### 4.2 Expression of AVT system

The AVT system (AVP in mammals) exerts physiological effects by increasing or altering water reabsorption mechanisms in kidneys and vasculature. AVP exerts its biological effects through binding to three AVP receptor subtypes, VR1a, VR1b, and VR2 receptors, that are members of the g protein coupled receptor superfamily (Dunzer et al., 2003). In birds, AVT and MT have been reported to be involved in oviposition and prolactin expression through binding to VR1a, VR1b, and VR2 receptors and MT receptors that are analogous to oxytocin receptors in mammals (Wu et al., 2019). In individually phenotyped broilers, both AVT and VR2 receptor expression were upregulated in the kidney of HWE compared to LWE (water inefficient) broilers, supporting an hypothesized role of enhanced renal water reabsorption contributing to greater water efficiency in the HWE line (Lassiter et al., 2025). In contrast, downregulation of hypothalamic AVT and VR2 in the HWE line were hypothesized to contribute to lower water intake exhibited in HWE compared to HWCR broilers (Aloui et al., 2023).

AVP was hypothesized to affect the small intestine by increasing water and salt secretion, or by direct interference with active Na^+^ transport (Soergel et al., 1968). AVP has been demonstrated to affect anion secretion in the colon indirectly through stimulation of glucagon-like peptide or peptide YY secretion from entoendocrine L-cells (Pais et al., 2016). In an ileal cell line, AVP induced growth promotion and proliferation through VR1a receptors and was hypothesized to play a role in the maintenance of the small intestinal mucosa by regulating intestinal epithelial cell proliferation and migration (Chiu et al., 2002). In the cat ileum, AVP increased superior mesenteric arterial pressure, while decreasing capillary filtration, and superior mesenteric venous pressure, indicating that ADH affected capillary hemodynamics by constricting arterioles in the mesenteric vasculature (Quillen et al., 1977). Continuous infusion of AVP decreased microvascular flow in the jejunum leading investigators to hypothesize that AVP may have adverse effects on intestinal oxygenation in pigs (Knotzer et al., 2005).

Although there were no differences in AVT expression in the gut segments due to line (Figure 2), AVT mRNA expression was induced by heat stress in the upper duodenum, lower duodenum, and cecum (Figure 3) and in the kidney (Lassiter et al., 2025). Arroyo et al. (2022) demonstrated that AVP is synthesized *de novo* and binds VR2 receptors that initiates the cAMP cascade that fuses vesicles containing AQP2 proteins to the apical membrane in collecting ducts to facilitate water reabsorption. Evidence of AVT mRNA expression induced by heat stress in the intestinal tract (Figure 3) suggests that local *de novo* production of AVT might be involved in water movement in intestines in heat-stressed broilers. In mammals, intestinal arterioles are particularly responsive and vasoconstrict in response to pM concentrations of AVP that binds to VR1a receptors (Altura, 1975; Liard, et al., 1982). Thus, increased AVT expression could play a role in the well-known thermoregulatory response in which blood flow is shunted from internal organs (e.g., splanchnic vasculature) to heat-dissipating organs such as the skin, wattle, and comb and the respiratory tract (Proppe, 1980; Wolfensen et al., 1981; 1982; Bottje W. G. and Harrison P. C., 1986; Bottje W. G. and Harrison P. C., 1986).

### 4.3 Expression of RAS system

The RAS system plays a significant role in gut physiology through; a) effects on motility (Robertson and Rubin, 1958; 1962), b) interactions of the RAS system with gut microbiota (Jaworska, et al., 2021), and c) the presence of all components (enzymes and receptors of the RAS system) throughout the intestine (see Zizzo and Serio, 2023). However, Zizzo and Serio (2023) also indicated that … *very little research has been devoted to the effective role and functional significance of RAS* (in the gut) *…* There is little information available on RAS system involvement in the intestinal tract in birds. An elegant study by Mueller et al. (2014) demonstrated that the RAS system is present and active in developing broiler embryos. Injection of captopril (an angiotensin converting enzyme [ACE] inhibitor), caused a reduction in mean blood pressure and injection of angiotensin II elevated blood pressure compared to controls. Captopril-induced inhibition of angiotensin II formation also decreased plasma osmolarity (Mueller et al., 2014). In the present study, there were few differences in intestinal RAS gene expression. Thus, it is difficult to develop a clear picture of the role of RAS in the intestines attributed to the phenotypic expression of water efficiency. Further investigation of the role of RAS in intestinal physiology in birds in general and broilers specifically is warranted. We have observed a sex difference in RAS expression with mRNA expression for AGT, REN, ACE, AT1 and AT2 being lower in females in the lower duodenum compared to males whereas expression of all five genes was higher in the cecum in females compared to males (unpublished observations).

### 4.4 Expression of AQP system and tight junction genes

The movement of water across gut epithelial cells occurs either by transcellular pathways (e.g., passive diffusion, aquaporin channels and transporters) or paracellular routes (regulated by tight junction proteins) (Zhu, et al., 2016). AQP proteins exhibit 13 isoforms that are grouped into those selectively permeable to water (AQP0-AQP8), those permeable to water, glycerol, and urea (AQP3, AQP7-AQP10), and ‘nonclassical’ ones (AQP11-APQ12) (Ishibashi, et al., 2009).

AQP1 mRNA expression in the upper duodenum was higher in HWE compared to MRB under both TN and HS conditions and there were both main effects attributed to line and temperature (Tables 2 and 3). Expression of AQP1 was elevated (P < 0.06) in the lower duodenum in response to HS (Table 6). In mammals, AQP1 has not been reported to be expressed in intestinal cells (Takata et al., 2004). However, AQP1 has been detected in lymphatic vessels located in the submucosa and lamina propria of rat intestines (Nielsen et al., 1995; Koyama et al., 1999) as well as in lymphatic endothelial cells (Ohtani et al., 2003). It was hypothesized that AQP1 could play a role in water movement from lymph fluid back into plasma (Takata et al., 2004). AQP1 is present in erythrocytes and responsible for 85% of water movement across red blood cell membranes (Mathai et al., 1996). While AQP2 was not identified in chicken intestines, AQP5 was detected in the jejunum, ileum, and colon (Ramirez-Lorca et al., 2006) and localized to mucous secreting cells in the duodenum (Ramirez-Lorca et al., 2006; Matsuzaki et al., 1999). In the present study, whole gut segments would contain not only intestinal mucosa, but serosa as well as lymphatics, blood vessels, and erythrocytes. Possibly, increased AQP5 expression would enhance hydration of mucus in the small intestine contributing to gut barrier integrity. These findings would suggest that there could be enhanced water movement (reabsorption) in HWE birds in the upper duodenum where the greatest volume of water reabsorption in the gut occurs.

AQP1, AQP3, and AQP5 were upregulated and AQP2 downregulated in HWE compared to MRB in the upper duodenum. The most notable differences in AQP and tight junction genes were observed in birds maintained under HS conditions. HS upregulated mRNA expression of AQP1, 3 & four in the upper duodenum, AQP1 (P < 0.06), AQP3, and AQP5 in the lower duodenum, and AQP3 and five in the cecum compared to broilers maintained under TN conditions. AQP3 was the only member of the AQP3 family that was upregulated by HS in all three gut segments.

A comprehensive study on tight junction expression in HWE and LWE broilers was reported (Greene et al., 2025). In that study, HWE birds exhibited significantly lower clearance of fluorescein isothiocyanate-dextran (FITC-D) which assesses water movement out of the gastrointestinal tract. Thus, LWE (water inefficient) broilers may have increased water loss into the gut lumen tract that would directly contribute to water inefficiency. In the present study, there were no differences in gene expression between HWE and MRB broilers at TN or HS temperatures in all three intestinal segments (Tables 3, 6, 7). The lack of effect of HS on mRNA expression for OCLN, CLDN2, and CLDN15 in the upper duodenum, and upregulation of all three genes in the lower duodenum, and CLDN2 and CLDN15 in the cecum contrasts with the report by Greene et al. (2025). A possible explanation for differences in findings could be due to the lines of broilers that were investigated in Greene et al. (2025), HWE and LWE in contrast to HWE vs. modern random bred (MRB) (the unselected broiler line) in the present study. Further investigation in this area is certainly warranted.

Considerable effort has been made in the investigation of the role of AQP3 in normal and pathophysiological conditions of the intestines in maintaining water transport, intestinal permeability, fluid secretion, and nutrient absorption (e.g., glucose, amino acids, lipids (Zhu et al., 2016; 2023; Ikarashi et al., 2019; Yde et al., 2021). Zhu et al. (2023) proposed that AQP3 could be a therapeutic target in diarrhea and constipation. Downregulation of AQP3 in intestinal epithelial cells would reduce water reabsorption whereas over-expression or upregulation would increase water reabsorption and constipation. In the present study, upregulation of AQP3 during heat stress in each of the gut segments could be associated with enhanced water reabsorption in birds undergoing heat stress. Water reabsorption (or prevention of water loss) would also be enhanced by upregulation of tight junction proteins during HS in the lower duodenum and cecum (Figure 3). It is interesting to note that AQP3 and AQP5 were upregulated in kidney of HWE compared to low water efficiency broilers (Lassiter et al., 2025).

## 5 Summmary

Water efficiency in the poultry industry is becoming increasingly important for sustainability. In this study, we investigated gene expression in the upper and lower duodenum and cecum in male broilers in response to heat stress in a base unselected line (modern random bred, MRB) and a line selected for water efficiency (HWE). As expected, HSP mRNA expression were higher in broilers subjected to heat stress in all three gut segments. HSP expression was higher in HWE compared to MRB broilers (main effect) with the exception of HSP70 in the lower duodenum. While this may suggest that HWE intestinal tissue was more susceptible to HS, elevations of HSPs in HWE during heat stress could provide increased cytoprotection and/or help maintain function of critical proteins. These mechanisms could be investigated in future studies. Heat stress induced AVT expression in all three gut segments. Although there was no main effect of line on AVT expression, a main effect of line was observed in VR2 expression in the upper and lower duodenum in the HWE group compared to expression levels in the MRB group. This implies that the AVT-VR2 expression could play a role in water homeostasis in the duodenum in the HWE broilers. HWE broilers exhibited upregulation of AQP1, AQP3, and AQP5 in the upper duodenum compared to the MRB group that could be hypothesized to lead to greater water reabsorption/retention.

The results of this study add to the fundamental molecular mechanisms associated with the phenotypic expression of water efficiency being developed by our group and how HWE broilers respond to heat stress. Additional mechanistic studies are needed to fully understand the roles that gene expression of these water homeostasis systems play in water efficiency and heat stress. The findings presented here add to other studies (Aloui et al., 2023; Greene et al., 2024; Lassiter et al., 2025; Santamaria, et al., 2025) that help paint an overall fundamental expression ‘landscape’ associated with biological water homeostasis. We want to point out that we can only discuss gene expression in this study in the context of whole segments of gut tissue as we did not separate mucosa from serosa nor investigate the dynamic nature of the gut; e.g., movement of cells from the crypt to villus tip, and the rapid turnover of cells that is well documented in the gut epithelial cells. The report by Aloui et al. (2023) investigated hypothalamic gene expression in male broiler progeny of HWE and LWE breeders which differs from the present study in which male broilers were investigated that were progeny of the base population MRB breeders compared to HWE breeders. Gut tissues in this study were obtained previously (Lassiter et al., 2025) that reported kidney expression of the same sets of genes as in the present study. The results do provide new insight into possible mechanisms responsible for water efficiency in these unique populations of broiler lines.

## Data Availability

The original contributions presented in the study are included in the article/supplementary material, further inquiries can be directed to the corresponding author.
